# Detailed mechanism and impact of new-onset late right heart failure during LVAD support

**DOI:** 10.1186/s13019-020-01300-1

**Published:** 2020-09-10

**Authors:** Teruhiko Imamura

**Affiliations:** Second Department of Internal Medicine, University of Toyama, 2630 Sugitani Toyama, Toyama, 930-0194 Japan

**Keywords:** Hemodynamics, Mechanical circulatory support, Central venous pressure, Continuous flow

## Abstract

Not applicable.

To the editor,

Left ventricular assist device (LVAD) unloads the left ventricle and increases cardiac output, whereas LVAD might not be beneficial or rather sometimes harmful for the right heart. New-onset late right heart failure is receiving great concern as unsolved comorbidity thus far. Wagner and colleagues demonstrated that preoperative right heart failure enhanced the risk of early right heart failure post-LVAD and persistent late right heart failure, but not the risk of new-onset late right heart failure [1]. Their findings let us hypothesizing different etiology between early and late right heart failures. Several concerns should more enhance the implication of their findings.

Their concept of “new-onset late right heart failure” would be almost similar to “late-onset right ventricular failure” that we proposed in 2014 [2]. I completely agree with them that a new onset late right heart failure would have a unique etiology, which cannot be predicted simply by the existence of preoperative right heart failure. Instead, as we hypothesized, considerable shrinkage of the left ventricle and associated positive remodeling of the right ventricle during LVAD supports might be a mechanism of late right heart failure [2]. Did any patients have preoperative small left ventricle or etiology of hypertrophic cardiomyopathy? How do the authors hypothesize the mechanism of late right heart failure?

Our team recently demonstrated that right heart failure was associated with hemocompatibility-related adverse events [3]. In their study, late right heart failure was associated with higher mortality. Do the authors have data on causes of death in their study? Such data would more clarify the clinical impact of late right heart failure during LVAD supports.

## Data Availability

No data.

## Abbreviations

LVAD: Left ventricular assist device
